# Optimizing Antioxidant and Biological Activities of *Quercus Fructus*: Synergistic Role of Inner Shell and Extraction Methods

**DOI:** 10.3390/antiox14080976

**Published:** 2025-08-08

**Authors:** Jin Gyeom Kim, Hajeong Kim, Beulah Favour Ortutu, Woochan Jeong, Su-In Yoon, Inhwa Han, Jin Ah Cho

**Affiliations:** 1 Department of Food and Nutrition, Chungnam National University, Daehak-ro 99, Yuseong-gu, Daejeon 34134, Republic of Korea; iigla2005@o.cnu.ac.kr (J.G.K.); gkwjd200@o.cnu.ac.kr (H.K.); ortutubeulah@o.cnu.ac.kr (B.F.O.); jwoochan12@gmail.com (W.J.); ihan@cnu.ac.kr (I.H.); 2 Glocal Life-Care Convergence Program, Interdisciplinary Education Center for the Innovative Next Generation Leaders in Glocal Lifecare, Chungnam National University, Daehak-ro 99, Yuseong-gu, Daejeon 34134, Republic of Korea; 3 Research Center for Microbiome-Brain Disorders, Chungnam University, Daehak-ro 99, Yuseong-gu, Daejeon 34134, Republic of Korea; suinyoon@cnu.ac.kr

**Keywords:** plant-derived bioactive, inner shell utilization, quercetin-7-glucoside

## Abstract

This study comprehensively evaluated how the inclusion of the inner shell and the choice of extraction method influence the antioxidant activity of *Quercus Fructus* (QF). Eight QF extracts were prepared with or without the inner shell using stirring (S) and ultrasonication (U) with 80% ethanol, boiled water (B) and autoclave (A) with distilled water. Among them, the ultrasonication extract with inner shell (IU) exhibited the highest antioxidant capacity, showing strong DPPH radical scavenging (228.8 mg TEAC/g), ABTS activity (162.9 mg TEAC/g), reducing power (380.9 mg TERP/g), and SOD-like activity (38.1%). HPLC-UV profiling identified quercetin-7-glucoside (Q7G) as a major detectable compound, although several polar phenolics remained unidentified. In LPS-stimulated Raw 264.7 cells, IU significantly suppressed nitric oxide production and iNOS expression without cytotoxicity. Additionally, IU treatment markedly reduced ROS accumulation in H_2_O_2_-exposed zebrafish embryos. These findings suggest that including the inner shell with ultrasonication extraction synergistically enhances QF’s antioxidant efficacy, suggesting a practical strategy for maximizing the functional potential of QF in natural antioxidant development.

## 1. Introduction

Excessive generation of reactive oxygen species (ROS) during cellular stress or injury can trigger inflammation and damage to cells and tissues. Thus, controlling ROS and the associated inflammatory response is crucial for maintaining homeostasis, and recent research has focused on natural functional foods with antioxidant and anti-inflammatory capabilities [1,2,3,4].

*Quercus Fructus* (QF), the fruit of commonly encountered oak tree species in Korea, exhibits diverse varieties, such as *Quercus serrata*, *Quercus dentata*, and *Quercus acutissima*. Although it has been used as a common culinary ingredient in Korea for centuries, it is one of the most promising natural resources due to its nutritional and medicinal properties [5,6,7]. QF consists of an outer shell, an inner shell, and a fruit positioned between them. QF is known for its astringent taste, attributed to plant-derived polyphenolic compounds such as gallic acid, ellagic acid, and gallotannin [8,9,10]. These compounds have demonstrated antioxidant and anti-inflammatory effects [11,12,13].

One underexplored aspect of QF utilization is the role of the inner shell. Recent studies reported that QF powder retains higher total polyphenol and flavonoid contents when the inner shell is included versus when only the fruit is used [7,14]. This suggests that the often-discarded inner shell may significantly contribute to the bioactive compound profile [5,15]. However, it remains unclear how inner shell inclusion influences the functional antioxidant efficacy of QF extracts, especially under different extraction conditions. Limited research has addressed the combined impact of plant tissue composition and extraction method on antioxidant outcomes in biologically relevant systems. Additionally, the choice of extraction method is known to greatly affect the yield and activity of phytochemicals [16,17]. Conventional and innovative extraction techniques, including stirring, ultrasonication, boiling, and autoclave extraction, which represent high-temperature and high-pressure conditions, can differentially isolate antioxidant compounds. To date, few studies have systematically examined how varying extraction processes modulate the antioxidant capacity of QF extracts, particularly in conjunction with inner shell inclusion. Furthermore, separating the inner shell from the fruit is labor-intensive in practice; if its inclusion proves beneficial, it could streamline processing and improve functional outcomes.

In this study, we addressed these gaps by systematically evaluating four different extraction methods—stirring extraction and ultrasonication extraction with 80% ethanol, boiling extraction and autoclave extraction with water—applied to QF powder either with the inner shell (both fruit and inner shell) or without (fruit only) in terms of antioxidant activity and its biological effects in vitro and in vivo. This multifaceted approach enabled us to determine an optimal extraction strategy and, for the first time, demonstrate that specific extraction conditions translate into superior antioxidant efficacy in living systems.

## 2. Materials and Methods

### 2.1. Extraction Preparation and Reagents

QF samples were collected on 5 October 2022, from the university forest of Chungnam National University (Daejeon, Republic of Korea), and fully matured fruits were selected. QF was divided into two groups: one with all shells removed, called fruit (F), and the other one only the outer shell removed, called inner shell + fruit (I). They were dried in a 55 °C food dryer for 3 h, followed by pulverization using a high-speed grinder in 30 cycles of 5 s each.

For ethanol extraction, 5 g of QF powder and 50 mL of 80% ethanol were mixed and stirred at 200 rpm for 30 min using a plate stirrer (HS1-A, LABTron, Seoul, Republic of Korea) at room temperature. This process was repeated three times to obtain the stirred extract (S). The same procedure was performed using an ultrasonic bath at room temperature and 40 kHz ultrasonication (WUC.A03H, DAIHAN SCIENTIFIC, Wonju, Korea) for 15 min, repeated three times to obtain the ultrasonication extracts (U). The choice of 80% ethanol as the extraction solvent was based on its similar polarity to methanol, while avoiding the latter’s toxicological concerns in food-related studies (Plaskova and Mlcek [18]). Gallic acid, a representative phenolic compound found in acorns, is known to have the highest solubility in methanol, followed by ethanol (Daneshfar et al. [19]). Thus, 80% ethanol offered a suitable balance between extraction efficiency and safety [8,18,19].

For water extraction, 5 g of crushed sample was added to 50 mL of distilled water. The boiling extract was prepared by boiling using a hot plate for 30 min (B). The autoclave extract was prepared at 121 °C and 0.135 MPa pressure for 15 min by autoclave (HB-506, Hanbaek Scientific Co., Bucheon, Republic of Korea) (A).

All eight extracts were then centrifuged at 27,000 rpm at 5 °C for 20 min using a centrifuge (Avanti JXN-30, Beckman Coulter, Brea, CA, USA), and the supernatant was collected. The concentration of all extracts was adjusted to 100 mg/mL [20,21,22]. For the in vitro experiments, the supernatant was filtered using a 0.25 µm filter before use. The extraction conditions were chosen based on prior studies. Stirring and ultrasonication conditions were applied according to the method described by Cho et al. [23], and the boiled water extraction was adopted from Hong et al. [24]. Autoclave extraction, following a standard sterilization protocol, was used to investigate the effectiveness of high-pressure heat treatment.

Gallic acid, 3,4-dihydroxybenzoic acid (3,4-DHBA), p-coumaric acid, Chlorogenic acid, caffeic acid, syringic acid, quercetin-7-O-glucoside (Q7G), 1,3,5-tri-O-caffeoylquinic acid (1,3,5-TCQA), luteolin, quercetin, apigenin, and kaempferol were purchased from Chengdu Biopurify Phytochemicals Ltd. (Chengdu, China). Catechin, epigallocatechin gallate (EGCG), myricetin-3-O-glucoside, and quercetin-3-O-glucoside were obtained from Extrasynthese SAS (Genay Cedex, France). All standard compounds had a purity of ≥95%. HPLC-grade acetonitrile, methanol, formic acid, Ferulic acid, quercetin, 2,2-diphenyl-1-picrylhydrazyl (DPPH), 2,2′-azino-bis (3-ethylbenzothiazoline-6-sulfonic acid) (ABTS), potassium persulfate, Folin–Ciocalteu’s reagent, sodium carbonate, sodium phosphate buffer, potassium ferricyanide, trichloroacetic acid, ferric chloride, pyrogallol, HCl, Trolox and 2′,7′-dichlorofluorescein diacetate (DCFH-DA) were purchased from Sigma-Aldrich (St. Louis, MO, USA). Diethylene glycol and NaOH were obtained from Daejung Chemicals & Metals Co. (Siheung, Republic of Korea). Lipopolysaccharide (LPS) was purchased from Invitrogen (San Diego, CA, USA), and hydrogen peroxide (H_2_O_2_) was purchased from Samchun Chemicals (Seoul, Republic of Korea).

### 2.2. HPLC-UV Analysis

Detection of polyphenolic compounds was performed at 280 nm. Compound identification was conducted by comparing retention times and UV absorption spectra with those of authentic standards. Quantification was carried out using calibration curves constructed from serial dilutions of standard compounds. A total of 16 flavonoids and phenolic acids were quantified: gallic acid, 3,4-dihydroxybenzoic acid (3,4-DHBA), catechin**,** chlorogenic acid, caffeic acid, syringic acid, epigallocatechin gallate (EGCG), myricetin-3-glucoside, p-coumaric acid, quercetin-7-glucoside, quercetin-3-glucoside, 1,3,5-Tri-O-caffeoyl quinic acid (1,3,5-TCQA), luteolin, quercetin, apigenin, and kaempferol. Analysis was performed using HPLC coupled with a UV detector (1260 Agilent, Santa Clara, CA, USA), and the separation was performed using an Agilent Zorbax Eclipse Plus C18 column (4.6 × 150 mm, 5-micron) maintained at 35 °C. The mobile phase consisted of solvent A (0.1% (*v*/*v*) formic acid in distilled water) and solvent B (0.1% (*v*/*v*) formic acid in acetonitrile solution). The flow rate was 0.8 mL/min.

The gradient elution program for flavonoid and phenolic acid separation was set as follows: 0–3 min, 0–5% B; 3–8 min, 5–10% B; 8–22 min, 10–30% B; 22–25 min, 30–70% B; and 25–27 min, 70–100% B. Subsequently, column washing was performed at 100% solvent B for 10 min, followed by re-equilibration with 5% solvent B to prepare for the next sequence.

### 2.3. Evaluation of Bioactive Compounds and Antioxidant Activity

#### 2.3.1. Determination of Total Polyphenol Content

Total polyphenol content was determined using the Folin–Ciocalteu method [25,26]. A 0.1 mL sample was mixed with 10 mL of 2% sodium carbonate solution. After 2 min, 0.1 mL of 50% Folin–Ciocalteu reagent was added. The mixture was allowed to react at room temperature for 30 min. Absorbance was measured at 750 nm using a UV-Vis spectrophotometer. Ferulic acid in 80% ethanol was used as the standard. Results were expressed as µg ferulic acid equivalents (FAE) per mL of extract (µg FAE/mL) [27,28]. All measurements were performed in triplicate, and the results are presented as mean ± standard deviation (SD). A sample blank was used to exclude the absorbance originating from the sample itself.

#### 2.3.2. Determination of Total Flavonoid Content

Total flavonoid content was determined by mixing 0.2 mL of sample with 2 mL of diethylene glycol and 0.2 mL of 1 N NaOH [29]. The mixture was incubated at 37 °C for 1 h. Absorbance was measured at 420 nm. Quercetin in 80% ethanol was used as the standard. Results were expressed as µg quercetin equivalents (QE) per mL of extract (µg QE/mL). All measurements were performed in triplicate, and the results are presented as mean ± standard deviation (SD). A sample blank was used to exclude the absorbance originating from the sample itself.

#### 2.3.3. 2,2-Diphenyl-1-picrylhydrazyl (DPPH) Radical Scavenging Activity

The antioxidant activity was evaluated using 100 µM DPPH in methanol [30]. A 0.5 mL sample was mixed with 5 mL of DPPH solution and incubated in the dark for 30 min. Absorbance was measured at 517 nm. Trolox in 80% ethanol was used as the standard. A sample blank was used to exclude the absorbance originating from the sample itself.

#### 2.3.4. 2,2′-Azino-bis (3-Ethylbenzothiazoline-6-Sulfonic Acid) (ABTS) Radical Scavenging Activity

An ABTS radical cation was generated by reacting 7 mM ABTS with 140 mM potassium persulfate in distilled water and allowing the mixture to stand in the dark for 12–16 h [31]. The solution was diluted with ethanol to obtain an absorbance of 0.7 ± 0.002 at 734 nm. For the assay, 150 µL of the sample was mixed with 3 mL of ABTS solution and allowed to react for 2.5 min [31,32]. Absorbance was measured at 734 nm. Trolox in 80% ethanol was used as the standard. A sample blank was used to exclude the absorbance originating from the sample itself.

#### 2.3.5. Reducing Power Assay

The reducing power was determined by mixing 100 µL of sample with 500 µL of 0.2 M sodium phosphate buffer (pH 6.6) and 50 µL of 1% potassium ferricyanide [33]. The mixture was incubated at 50 °C for 20 min, followed by the addition of 2.5 mL of 10% trichloroacetic acid. After centrifugation at 1000× *g* for 10 min, 500 µL of the supernatant was mixed with 500 µL of distilled water and 100 µL of 1% ferric chloride. Absorbance was measured at 700 nm. Trolox in 80% ethanol was used as the standard. A sample blank was used to exclude the absorbance originating from the sample itself.

#### 2.3.6. Superoxide Dismutase (SOD)-like Activity

To assess SOD-like activity, 100 µL of sample was mixed with 1.5 mL of 50 mM Tris-HCl buffer (containing 10 mM EDTA, pH 8.0) and 0.1 mL of 7.2 mM pyrogallol [34]. The mixture was incubated at 25 °C for 10 min. The reaction was stopped by adding 0.5 mL of 0.1 N HCl, and the absorbance was measured at 420 nm. A sample blank was used to exclude the absorbance originating from the sample itself.

### 2.4. Cell Culture, Viability Assay, and Imaging Assays

Mouse macrophage cells (Raw 264.7) were purchased from the American Type Culture Collection (Manassas, VA, USA). The cells were cultured in Dulbecco’s Modified Eagle’s medium (DMEM; Thermo Fisher Scientific, Waltham, MA, USA) with 1% Penicillin-Streptomycin (Sigma-Aldrich Co., Saint louis, MO, USA) and 10% heat-inactivated fetal bovine serum (FBS) (Thermo Fisher Scientific) at 37 °C under an atmosphere containing 5% CO_2_.

For the cell viability assay, Raw 264.7 cells were seeded in 96-well plates and treated with various concentrations of QF extracts (12.5, 25, 50, 100, 200, 400, 800 µg/mL) for 48 h. Viability was examined using an EZ-Cytox WST assay kit according to the manufacturer’s instructions (Daeil Lab Service Co., Ltd., Seoul, Republic of Korea). Absorbance was measured at 450 nm using a Microplate Spectrophotometer (xMark™, Bio-Rad, Hercules, CA, USA). The cell viability rate was calculated based on the untreated control group.

For live cell imaging, Raw 264.7 cells were seeded in glass-bottom 6-well plates (P06-1.5H-N) and treated with QF extracts (25 μg/mL) for 48 h. After 24 h, LPS (1 μg/mL) was added, followed by an additional 24-h incubation. Time-lapse holotomography (HT) imaging was performed for 24 h using the HT-X1™ system (Tomocube Inc., Daejeon, Republic of Korea), generating four-dimensional (3D + time) datasets. The acquired 4D HT data were analyzed using the ‘(3D) single cell analysis’ pipeline in Tomoanalysis™ 2.0 software (Tomocube Inc.), which quantifies cell volume (μm^3^) and surface area (μm^2^). The analysis procedure was performed with reference to the TomoAnalysis™ 2.1 User Manual provided by the manufacturer. Measurements from the 24 h timepoint were compared to baseline (0 h) to calculate fold changes. Data were normalized to the mean of the negative control group, which was set to 1.

### 2.5. Nitric Oxide (NO) Assay

The amount of nitrite produced by cytokines in the culture medium was measured using Griess reagent (Promega Co., Madison, WI, USA) according to the manufacturer’s instructions using an xMark™ Microplate Spectrophotometer. The amounts of NO produced were calculated using a NO reference standard curve.

### 2.6. RNA Extraction & Reverse Transcription Polymerase Chain Reaction (RT-PCR)

Raw 264.7 cells were seeded in 6-well plates and treated with various concentrations of QF extracts (12.5, 25, 50 µg/mL) for 48 h. After 24 h, 1 µg/mL of LPS was added and the mixture was incubated for an additional 24 h. Cells were then homogenized using TRI reagent (MRC Inc., Cincinnati, OH, USA) for RNA extraction. Chloroform (Junsei Co., Tokyo, Japan) was added, and the homogenate was centrifuged at 12,000× *g* for 15 min at 4 °C. The supernatant was collected, isopropanol (Duksan Co., Ansan-si, Republic of Korea) was added, and the mixture was centrifuged at 12,000× *g* for 8 min at 20 °C. The supernatant was removed, and the RNA concentration from the pellet was quantified using a NanoDrop spectrophotometer (Thermo Scientific Inc., Waltham, MA, USA). The cDNA was synthesized using an RT Kit (Biofact Co., Daejeon, Republic of Korea). To quantify the mRNA expression of iNOS, RT-PCR was performed using 2 × Taq Basic PCR Master Mix according to the manufacturer’s instructions (Biofact Co., Daejeon, Republic of Korea) with the following gene-specific primer: iNOS (FW: 5′-AAT GGC AAC ATC AGG TCG GCC ATC ACT-3′, RV: 5′-GCT GTG TGT CAC AGA AGT CTC GAA CTC-3′). The amplification products were loaded onto 1.5% or 2% agarose gel for electrophoresis and observed using a gel documentation system (AE-9000 E-Graph, ATTO Co., Tokyo, Japan) under UV light.

### 2.7. Zebrafish Embryo Collection & ROS Measurement

Zebrafish (*Danio rerio*) embryos were supplied by the Zebrafish Center for Disease Modeling (ZCDM, Korea) and maintained in a temperature-controlled room at 28 °C with a 14:10 h day/night cycle. Zebrafish were fed brine shrimp 4 times per day. Experiments were performed following the Animal Research Guidelines at Chungnam National University (202410A-CNU-203).

The embryo toxicity test was conducted as a preliminary screening experiment for the subsequent ROS assay [35,36]. At 2 to 52 h post fertilization (hpf), zebrafish embryos were isolated in 96-well cell culture plates (Hyundai Micro Co., Seoul, Republic of Korea) at 1 embryo/well in egg water supplemented with 0.1% methylene blue and maintained in a temperature-controlled room at 28 °C. The embryos were then treated with QF (0, 100, 200, 400, 800 µg/mL) for various times as indicated. Embryos (*n* = 9 per group) were examined using an optical microscope (TM-10S, Taeshin Bioscience, Gyeonggi-do, Republic of Korea).

For ROS measurement, we revised the present protocol for our experiment setting [36]. The number of embryos per group for ROS measurement was determined based on statistical analyses from a prior experiment using the IU extract. In this study, 10–12 embryos per group were used, and the antioxidant effect of QF against H_2_O_2_-induced ROS generation was found to be large to very large (Cohen’s d = 1.252 to 2.867). G*Power 3.1.9.7 analysis indicated that such effect sizes would require a minimum of 5 embryos per group (Cohen’s d = 2.867) to achieve a statistical power of 0.8 (α = 0.05, two-tailed). To minimize unnecessary animal use, the IU experiment was terminated early once sufficient statistical significance was achieved. Based on these results, the subsequent FU experiment was conducted using only 5 embryos per group. All experimental designs were guided by the principles of the 3Rs (Replacement, Reduction, Refinement) to minimize animal use while maintaining statistical validity

Zebrafish embryos at 2 hpf were isolated in 96-well plates at 1 embryo/well in egg water supplemented with 0.1% methylene blue and treated with QF (0, 12.5, 25, 50, 100, 200 µg/mL) for 24 h. Then, 5 mM H_2_O_2_ was added, and the mixture was incubated for an additional 24 h to induce ROS. After incubation, the embryos were washed with egg water (*n* = 5–12 per group). At 48 hpf, the eggs were treated with egg water containing DCFH-DA (20 µg/mL), incubated for 1 h at 28 °C in the dark, and then washed with egg water. Images of stained embryos were observed using a digital microscope (Dino-Lite Digital Microscope, ANMO Electronics Co., New Taipei City, Taiwan) and analyzed by ImageJ 1.54k software.

### 2.8. Statistical Analysis

Data were analyzed using the SPSS 24.0 (SPSS Inc., Chicago, IL, USA) software. Results are displayed as mean ± standard deviation. Statistical analysis comparing two groups was performed using an independent t-test, and one-way analysis of variance (ANOVA), followed by Duncan’s multiple range tests, which were used to analyze two or more groups. Statistical significance was set at *p* < 0.05.

## 3. Results

### 3.1. HPLC-UV Analysis of QF Extracts

In this study, we prepared eight crude QF extracts from different extraction methods: fruit with inner shell using stirring (IS), fruit only using stirring (FS), fruit with inner shell using ultrasonication (IU), fruit only using ultrasonication (FU), fruit with inner shell using boiled water (IB), fruit only using boiled water (FB), fruit with inner shell using autoclave (IA) and fruit only using autoclave (FA).

**Figure 1:**
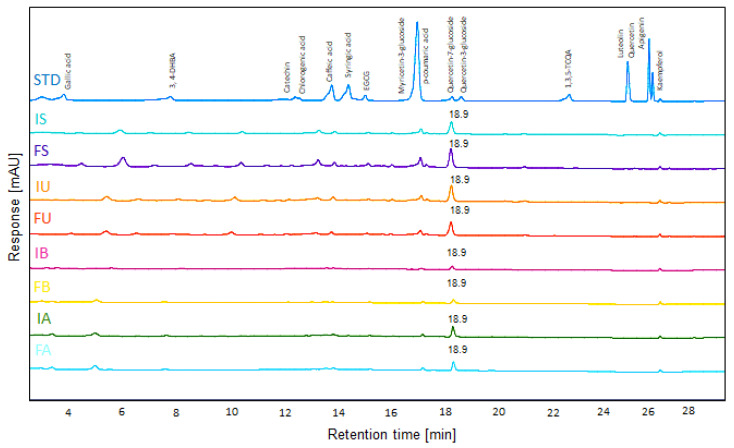
HPLC-UV (280 nm) chromatograms of QF extracts prepared by different extraction methods. Sixteen antioxidants were detected using HPLC/UV 280 nm (STD). Each line shows the standard alongside different 15 mg/mL QF extracts; I: inner shell with Fruit, F: fruit, S: stirring, U: ultrasonication, B: boiled water, A: autoclave.

HPLC-UV analysis was performed to detect several antioxidant factors. The presence of quercetin-7-glucoside (Q7G), a bioactive flavonoid, was confirmed by HPLC-UV analysis (Figure 1). A comparison with a standard compound confirmed an identical retention time (18.9 min), validating the presence of Q7G in QF. After validating the presence of Q7G in QF, we established a calibration curve for quantification, confirming a high linearity with an R^2^ value of 0.9996. Based on this, a quantitative analysis was performed, and the concentration of Q7G in each extract was determined as follows: IS (64.1 µg/mL), FS (93.6 µg/mL), IU (79.9 µg/mL), FU (67.2 µg/mL), IB (12.4 µg/mL), FB (14.1 µg/mL), IA (41.2 µg/mL), and FA (34.2 µg/mL). Q7G was most abundant in ethanol extracts (S, U) (Figure 1). Its selective enrichment in specific extracts indicates that specific extraction techniques can enhance the functional quality of QF by optimizing Q7G yield. Therefore, the presence and relative abundance of Q7G may be one of several contributing factors to the observed bioactivities of these extracts.

### 3.2. Contents of Phenolic and Flavonoid Compounds in Different QF Extracts

**Figure 2:**
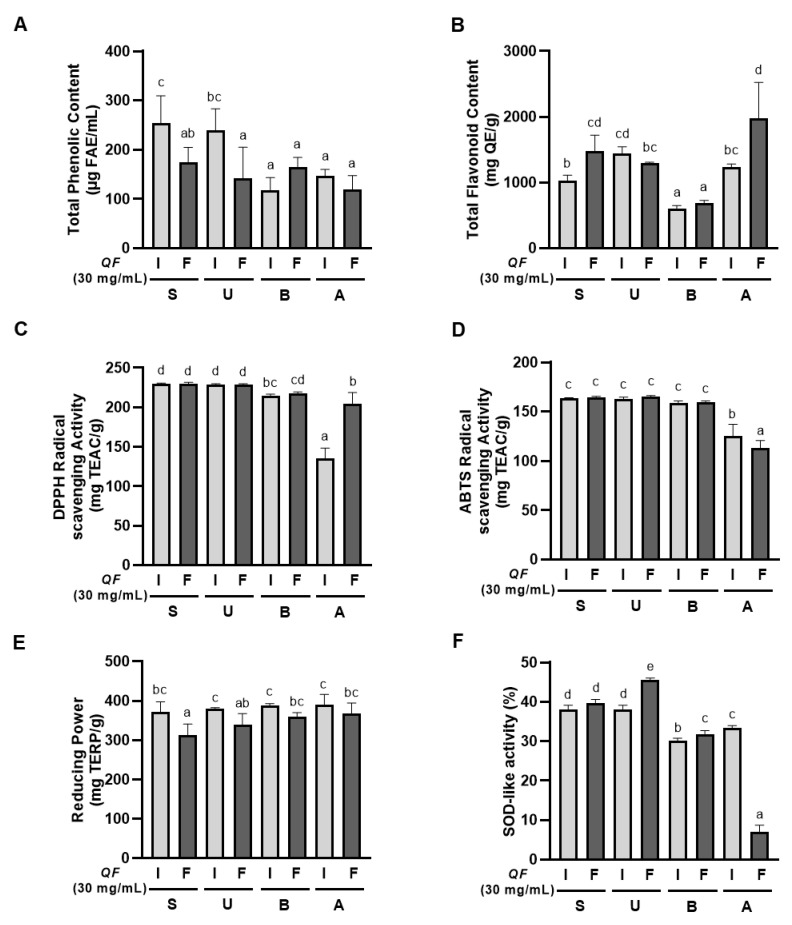
Colorimetric analysis of antioxidant activities of QF extracts (30 mg/mL) by extraction methods. Antioxidant activity of 30 mg/mL extracts from the different extraction methods were measured. (**A**) total phenolic content, (**B**) total flavonoid content, (**C**) 2,2-diphenyl-1-picrylhydrazyl (DPPH) assay, (**D**) 2,2′-Azino-bis (3-ethylbenzothiazoline-6-sulfonic acid) (ABTS) assay, (**E**) reducing power, and (**F**) superoxide dismutase (SOD)-like activity were measured. Results were represented as mean ± standard error with indication of statistical significance when *p*-value is less than 0.05. The letters are used for significant difference in ANOVA followed by Duncan’s post hoc test comparing all eight extraction methods (I: inner shell with Fruit, F: fruit, S: stirring, U: ultrasonication, B: boiled water, A: autoclave; FAE: Ferulic acid equivalent, QE: Quercetin equivalent, TEAC: Trolox equivalent antioxidant capacity, TERP: Total extractable resinous polyphenols).

After HPLC-UV analysis, we used 30 mg/mL QF extracts to compare the phenolic contents of the extracts. We discovered that the inner shell containing QF extract (I) has significantly higher total phenolic content compared to fruit-only QF extract (F) among ethanol extractions (S, U), while there was no significant difference between I and F in the water extraction methods (B, A) (Figure 2A). IS showed the highest total phenolic content among all eight extracts, followed by IU, both of which are ethanol-based extracts containing the inner shell.

The total flavonoid content showed that FS, IU, and FA extracts had the highest levels, with these three showing comparable results (Figure 2B). The pattern for total flavonoid content was more complex than total phenolic content, with no consistent trend being observed regarding inner shell inclusion across different extraction methods. The water boiling extraction method (B) showed the lowest flavonoid content among all methods.

In our results, ethanol extraction methods (S, U) consistently showed superior DPPH radical scavenging activity compared to water extraction methods, with autoclave extraction (A) showing the poorest performance (Figure 2C). Among the autoclave extracts, FA had higher DPPH scavenging activity than IA.

The ABTS radical scavenging activity results showed that stirring (S), ultrasonication (U), and boiling (B) methods performed similarly well, while autoclave extraction (A) showed significantly lower activity (Figure 2D). Interestingly, among the autoclave extracts, IA demonstrated higher ABTS activity than FA, which was opposite to the DPPH results.

In our study, ‘I’ had significantly higher reducing power than ‘F’ under all extraction conditions (Figure 2E). This pattern demonstrates that inner shell inclusion enhances the reducing power regardless of the extraction method used, suggesting that reducing power is closely related to the presence of compounds specifically found in the inner shell.

SOD-like activity was significantly higher in ethanol extraction methods (S, U) compared to water extraction methods (B, A) (Figure 2F), indicating that ethanol extraction is particularly effective for extracting compounds responsible for superoxide scavenging activity.

In summary, the antioxidant capacity of QF extracts varied significantly depending on both the extraction method and the inclusion of the inner shell. Ethanol extraction methods (S, U) consistently outperformed water extraction methods (B, A) in most assays, particularly for SOD-like activity. Inner shell inclusion (I) consistently enhanced reducing power across all extraction methods and improved total phenolic content in ethanol extractions. Among all extracts, IS and IU showed the most promising overall antioxidant profiles, combining high phenolic content with superior radical scavenging and reducing activities.

### 3.3. Morphology Changes of Cells by QF Extracts

**Figure 3:**
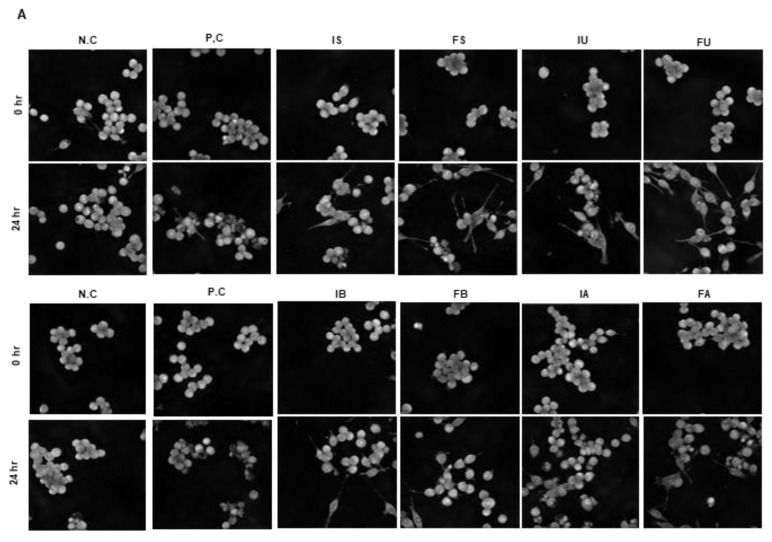

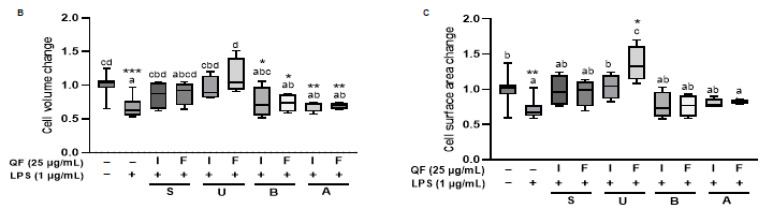
Evaluation of cell morphological changes by QF extracts using holotomography. Live images were recorded for 24 h in real time. The picture of the changes in the final cell volume and surface area after 0 and 24 h were taken (**A**) and analyzed using Tomoanalysis 2.1 software (**B**,**C**). Results as mean ± standard error with indication of statistical significance at different levels. * *p* < 0.05, ** *p* < 0.01, *** *p* < 0.001; * denotes independent *t*-tests performed between negative control and experimental group. The letters are used to denote levels of significance with ANOVA followed by Duncan’s post hoc test comparing all eight extraction methods, where groups not sharing a letter indicate statistical significance with *p* < 0.05 (N.C: negative control, P.C: positive control, I: inner shell + Fruit, F: fruit, S: stirring, U: ultrasonication, B: boiled water, A: autoclave).

Activation of Raw 264.7 cells by LPS treatment triggers an inflammatory response through the release of cytokines. Activated Raw 264.7 cells exhibit an irregular and rough morphology, with increased spreading and the formation of pseudopodia, which are extensions of the cell membrane used for engulfing particles during phagocytosis, which is accompanied by an increase in cell volume. If the inflammatory response persists excessively after activation, cell death occurs, resulting in decreased cell volume and surface area. To investigate the morphological effects of the extract at a non-cytotoxic concentration (25 µg/mL), which was determined by preliminary results (Supplementary Figure S1A,B), we recorded images before and after LPS treatment, and then examined the changes in cell volume over 24 h in real time (Figure 3A). To quantify the data on cell changes, we utilized the Tomoanalysis software 2.1. Using the negative control as a baseline value of 1, we compared cell volume and surface area changes across groups (Figure 3B,C). Our findings indicate that the cell volume and surface area of those treated solely with LPS significantly decreased after 24 h compared to untreated cells. However, in cells treated with both LPS and the IS, FS, IU FU extracts, there was no significant decrease in cell volume compared to the control cells, suggesting potential inhibition of LPS-induced cell death [37]. However, in water extracts such as IB, FB, IA, and FA, a significant decrease in cell volume was observed compared to the control, suggesting that water extracts have limited potential to prevent LPS-induced cell death. These results are similar to our WST assay. Moreover, we discovered that FU showed a higher cell surface area compared to IU and the negative control. As a result, FU appears to prevent LPS-induced cell death, but shows limitations in suppressing excessive cell activation. In addition, water extracts showed limited effects in preventing LPS-induced cell death.

### 3.4. Antioxidant Effect of Different QF Extracts

**Figure 4:**
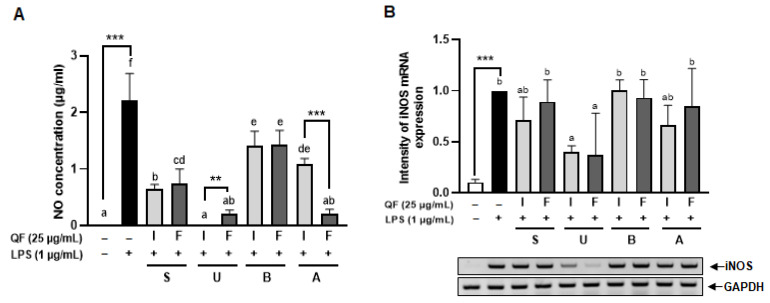
Inhibitory effects of QF extracts on NO concentration and iNOS expression. NO concentration was measured using NO assay (**A**). iNOS mRNA levels were measured by RT-PCR (**B**). GAPDH was used for loading the control. Graphic images were processed and analyzed with ImageJ to quantify the data, and the results are presented in graphical form. Results represent as mean ± standard error with indication of statistical significance when the *p*-value is less than 0.05. Additionally, symbols are used to denote levels of significance: (** *p* < 0.01; *** *p* < 0.001; Asterisk (*) denotes independent *t*-tests performed between values marked with line; over the bar graphs letter means results of ANOVA followed by Duncan’s post hoc test comparing all eight extraction methods, not sharing a letter indicate statistical significance with *p* < 0.05; I: inner shell + Fruit, F: fruit, S: stirring, U: ultrasonication, B: boiled water, A: autoclave).

Inducible nitric oxide synthase (iNOS) is an enzyme that synthesizes NO from l-arginine. While NO generated by iNOS plays various roles in both physiological and pathophysiological aspects, excessive production of NO can lead to oxidative stress [38]. Therefore, this study aimed to investigate whether the treatment with extract can reduce the increased expression of inducible nitric oxide synthase (iNOS) induced by LPS or decrease the already generated NO, thus confirming its antioxidant function. To compare the antioxidant efficacy based on extraction methods, QF were treated to Raw 264.7 cells at 25 μg/mL, followed by 1 μg/mL of LPS 24 h later. The amount of NO produced was assessed by examining the presence of NO in the media 24 h after LPS treatment. Cells treated with LPS only showed an increase in NO production compared to untreated cells (Figure 4A). Conversely, when treated with the extract, a concentration-dependent decrease in LPS-induced NO production was observed compared to cells treated with LPS only. In addition, there was a significant difference in NO concentration between the IU and FU, and between IA and FA after extraction treatment, suggesting that extracts containing the inner shell might exhibit different NO inhibition functions depending on the extraction method. These results demonstrated that QF inhibits NO production in Raw 264.7 cells at different levels based on the extraction method and the presence of the inner shell.

Further investigation into the expression of iNOS, the enzyme responsible for NO synthesis, was conducted through RT-PCR to compare mRNA expression levels based on QF methods. The 25 μg/mL extracts IU and FU showed a reduction in iNOS expression (Figure 4B).

### 3.5. Cytotoxic and Inhibitory Effects of QF on ROS Production in Zebrafish Embryos

**Figure 5:**
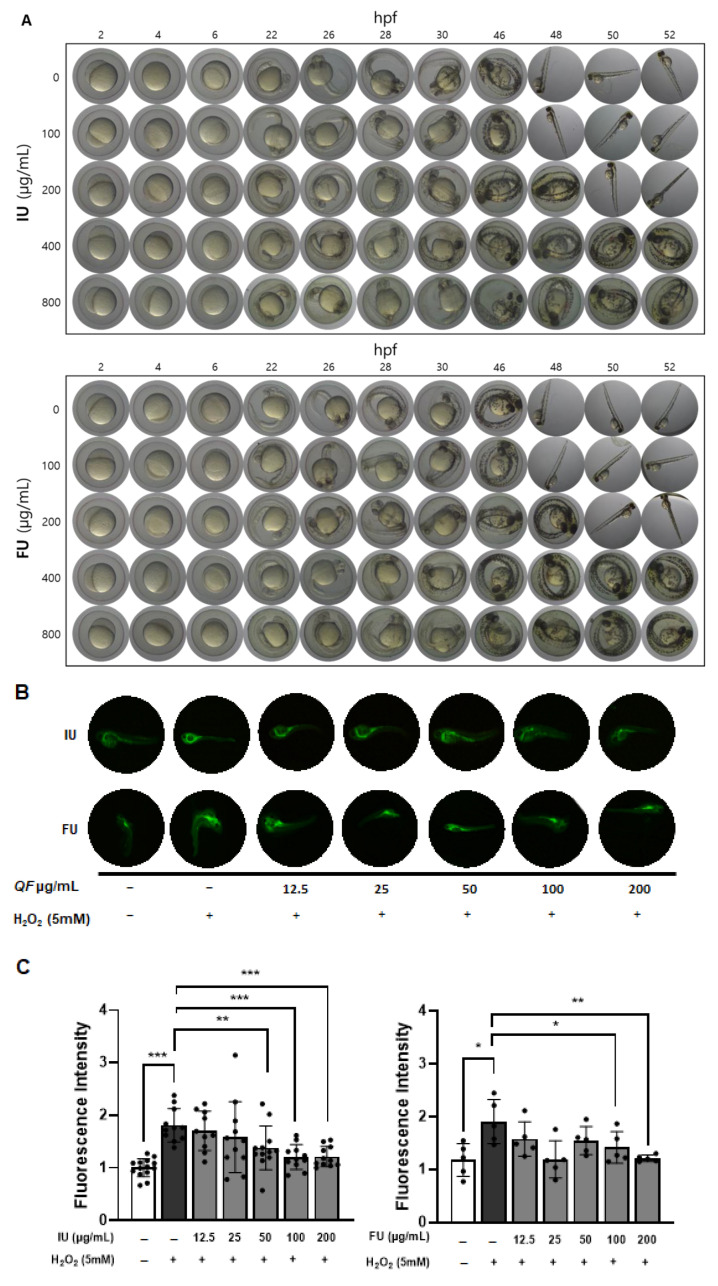
Antioxidant effects of QF extracts in H_2_O_2_-treated zebrafish embryos. Zebrafish embryos were incubated with QF in egg water for the indicated time (**A**). Zebrafish embryos were treated with QF extracts for 24 h and 5 mM H_2_O_2_ for an additional 24 h (**B**). Intensity of fluorescence was analyzed using ImageJ software (**C**). The asterisks are used to denote levels of significance using independent *t*-test: * *p* < 0.05, ** *p <* 0.01, *** *p* < 0.001; I: inner shell + Fruit, F: fruit, U: ultrasonication.

To evaluate the in vivo antioxidant activity of QF extracts, we used zebrafish models treated with ‘U’ extracts prepared from QF containing both fruit and inner shell or the fruit only, as our results above showed that ‘U’ extracts produced the greatest reduction in NO production. Initially, zebrafish embryos were treated with IU and FU extracts at concentrations of 0, 100, 200, 400, and 800 μg/mL as shown in Figure 5A. There were no difference between IU and FU regarding hatching time. In addition, 100 μg/mL QF had the same hatching time (48 h) as the negative control. However, the hatching time was delayed in the presence of both IU and FU extract at 200 μg/mL and above. In addition, no noticeable developmental delays or morphological abnormalities were observed at concentrations ≤ 200 μg/mL. However, at concentrations ≥ 400 μg/mL, most embryos exhibited severe development delays, resulting in postponed hatching. Based on these findings, the maximum concentration for both IU and FU extracts was determined to be 200 μg/mL, which was subsequently used in the following antioxidant experiments.

For the ROS inhibition ability of QF extracts, the IU-treated groups showed significant suppression of ROS levels at concentrations over 50 μg/mL compared to the H_2_O_2_-only group. In the FU-treated groups, a similar trend was noted, although ROS reduction became evident only at concentrations of 100 μg/mL or higher (Figure 5B,C).

These results indicate that both types of QF extracts can effectively suppress ROS production under oxidative stress in zebrafish embryos. However, the IU extract exhibited markedly superior antioxidant activity, as it significantly reduced ROS levels even at a lower concentration (50 μg/mL), whereas the FU extract required a higher dose to achieve comparable effects. This enhanced efficacy observed in the IU extract is likely attributable to its higher total phenolic content and the presence of Q7G, potentially alongside other co-extracted bioactive compounds, as confirmed by HPLC analysis.

## 4. Discussion

While previous studies have investigated QF’s antioxidant properties [39,40,41], our research provides the first comprehensive comparison of inner shell inclusion across multiple extraction methods, revealing previously unreported functional benefits. The combination of ultrasonication extraction with inner shell inclusion (IU) emerged as the optimal approach, demonstrating superior antioxidant activity through enhanced polyphenol extraction and preservation of bioactive compounds.

Previous research has established that ethanol extraction generally yields higher antioxidant activity than water-based methods for QF [6,42,43], which is consistent with our findings where ethanol extracts (IS: 254.27 μg FAE/mL, IU: 239.66 μg FAE/mL) showed superior total phenolic content and NO inhibition compared to water extracts. Our identification of Q7G as the primary detectable flavonoid, known for its enhanced bioavailability and iNOS inhibitory properties [44], provides mechanistic insight into the observed antioxidant effects, although the superior biological activity of ultrasonication extracts suggests involvement of additional co-extracted compounds.

Our findings suggest that Q7G may play a crucial role in reducing NO levels and enhancing antioxidant defense, reinforcing its potential as a key bioactive compound in QF extracts. This study underscores the significance of Q7G as a more effective functional ingredient compared to traditional phenolic acids, paving the way for further research into its therapeutic applications.

To ascertain the antioxidant effects of QF, oxidative stress induction was performed using LPS. We observed differences in antioxidant effects depending on the presence of the inner shell and the extraction method, as indicated by NO inhibition. The 25 μg/mL extracts were effective in decreasing iNOS expression. This is in agreement with Youn et al. (2016), whose study showed higher antioxidant activity at 50 μg/mL in *Quercus acutissima Carruth*, although the extraction methods and methods of assessing antioxidant activities were different from those of our study [12]. There might have been variations in the concentration of extracts causing a decrease in iNOS expression due to different extraction methods. The antioxidant activity of plant materials generally originates from their polyphenolic compounds. Several studies have shown a direct relationship between phenolic content and antioxidant activity of *Quercus* species [10]. As expected, the high antioxidant activity in this paper can be attributed to the phenolic content. These results indicate that different extraction methods may yield different mechanisms of action in antioxidant function.

The differential performance across four antioxidant evaluation methods reflects their distinct measurement mechanisms and target radical species. DPPH assays, utilizing nitrogen-centered radicals in organic solvents [30], showed ethanol extracts (S, U) consistently outperforming other methods, with water boiling (B) showing slightly lower activity and autoclave (A) extracts demonstrating the poorest performance, particularly IA < FA. ABTS assays with water-soluble cationic radicals [32] revealed that stirring, ultrasonication, and boiling methods (S, U, B) performed similarly well, while autoclave extracts showed lower overall activity but with a reversed pattern (IA > FA) compared to DPPH results. This discrepancy suggests that while autoclave processing generally reduces antioxidant capacity, it generates different antioxidant components in inner shell versus fruit extracts, with the former being more effective against ABTS radicals. The reducing power assay evaluates the electron-donating ability of antioxidants, independent of radical scavenging mechanisms [45], consistently favoring inner shell-containing extracts across all methods, indicating that tannins and other multi-hydroxyl compounds in the inner shell contribute significantly to the total reducing capacity. SOD-like activity, which assesses the ability of antioxidants to mimic superoxide dismutase by scavenging superoxide anion radicals [34], showed the most dramatic differences, with ethanol extracts (S, U) substantially outperforming water extracts (B, A), and demonstrating over 3-fold higher activity compared to FA extracts, highlighting the critical role of extraction solvent in preserving superoxide-scavenging compounds.

The superior performance of ethanol extracts (S, U) compared to water extracts (B, A) can be attributed to fundamental differences in solvent polarity and extraction conditions [46,47,48,49]. Organic solvents such as 80% ethanol possess higher solubility for polyphenols and flavonoid compounds than water, enabling the extraction of a broader spectrum of antioxidant components while maintaining relatively low temperatures that preserve thermolabile compounds. Conversely, water-based extraction methods, particularly autoclave processing under high temperature and pressure, can enhance release of bound phenolic acids through cell wall destruction but simultaneously cause structural degradation of heat-sensitive flavonoid glycosides [46]. The superiority of ultrasonication over stirring with identical solvent conditions demonstrates the additional benefit of acoustic cavitation in disrupting cellular microstructures and promoting rapid compound release without thermal degradation. These findings demonstrate that comprehensive antioxidant evaluation requires multiple assays to capture the full spectrum of protective mechanisms, while extraction optimization must balance compound release with preservation.

In the antioxidant component analysis using HPLC, only Q7G was clearly detected among the 16 tested phenolic standards, while colorimetric measurements showed significant differences in total phenolic and flavonoid contents between samples. This discrepancy indicates that the complex phytochemical diversity in QF extracts, including numerous unidentified compounds, contributes to the total antioxidant capacity. Q7G was detected at high concentrations in ethanol extracts containing only fruit (FS: 93.6 μg/mL) and IU (79.9 μg/mL). However, the lower Q7G concentration in the IU extract, which exhibited superior biological activity, suggests the possibility of synergistic effects among multiple components. Particularly intriguing was the FA extract’s high total flavonoid content (comparable to FS and IU) despite lower Q7G levels, suggesting that autoclave processing induces glycoside hydrolysis or structural modifications that reduce HPLC-detectable Q7G while creating degradation products reactive with colorimetric reagents. The inner shell’s primary contribution appears to be non-flavonoid polyphenols such as tannins, which explains the consistent enhancement of total phenolic content and reducing power in I-containing extracts. This compositional complexity underscores why extraction optimization cannot rely solely on individual compound quantification but must consider total bioactive profiles and their functional interactions.

The integration of in vitro cell culture and in vivo zebrafish studies provided robust validation of our biochemical findings. Raw 264.7 macrophage experiments revealed that ethanol extracts, particularly IU, effectively inhibited LPS-induced NO production and iNOS expression at 25 μg/mL, while water extracts showed limited or adverse effects. The holotomography analysis offered unique insights into cellular protection mechanisms, showing that extract treatment prevented LPS-induced cell volume reduction and death rather than typical inflammatory activation. The high LPS concentration (1 μg/mL) likely induced rapid cytotoxicity before classical morphological changes could manifest, making cell survival the primary measurable outcome. Zebrafish embryo studies confirmed these protective effects in a complex biological system, with IU extract demonstrating superior ROS suppression at lower concentrations (50 μg/mL) compared to the FU extract (100 μg/mL). This dose-dependent efficacy directly correlated with total phenolic content and Q7G concentrations, validating our biochemical measurements in a physiologically relevant context. The consistency between in vitro and in vivo results strengthens the evidence for bioactive compound stability and functional preservation across biological systems.

This study investigated the effects of the combination of inner shell inclusion and extraction methods on the antioxidant activity of QF from various perspectives, but the following limitations exist. First, in this study, HPLC-UV analysis was performed on a total of 16 standard compounds, and the detection wavelength was set to 280 nm. However, this condition may not be suitable for detecting hydrolysable tannins such as ellagitannins. In fact, several unresolved peaks were observed in the chromatogram of this study, suggesting the possible presence of polar polymeric compounds such as ellagitannins. However, since this study did not include standard materials for these compounds and did not perform LC-MS/MS analysis for structural confirmation, the exact identity of these compounds could not be determined. Therefore, future studies should combine LC-MS/MS-based structural analysis and multi-wavelength detection to clarify the presence of phenolic compounds not identified in the current analysis and their role in the antioxidant mechanism.

Second, although this study confirmed strong antioxidant activity through in vitro and in vivo models, there were limitations in quantitatively separating and analyzing the contributions of individual components such as Q7G and tannins due to the nature of the complex extract. Interactions between various bioactive substances (synergistic effects) and complex physiological activities may occur, which can hinder the clear distinction of the effects of specific individual components. Future studies should clarify the contribution of each component through fractionation extraction or component separation and recombination tests.

Third, in vivo experiments using zebrafish are useful models for confirming systemic biological effects, but they have limitations in terms of physiological similarity to humans. Therefore, follow-up studies using mammalian models such as mice are needed to validate and generalize these results. In addition, as this study adhered to the 3Rs principle (Replacement, Reduction, Refinement) to minimize animal use, the relatively small sample size per group (*n*) represents a limitation that should be considered when interpreting the data.

The greatest strength of this study is that it did not merely compare solvents but comprehensively analyzed the combination of inner shell inclusion and extraction method as key factors determining the antioxidant efficacy of QF extract. QF are raw materials that exist in a state where the shell and inner shell are mixed, and the process of removing the inner shell in actual industrial settings or processing can be cumbersome and inefficient. Therefore, one of the main objectives of this study was to experimentally clarify the validity of whether it is necessary to remove the inner shell and whether including the inner shell could be advantageous in terms of antioxidant activity.

To achieve this objective, this study applied four different extraction methods and systematically compared antioxidant activity based on whether the inner shell was included or removed. Notably, the highest antioxidant activity was observed under the ultrasonic extraction + inner shell (IU) condition. This finding is significant not only because it goes beyond simple chemical metrics but also because it is supported by biological activity through in vitro and in vivo analyses using cell and zebrafish models.

Additionally, HPLC analysis detected Q7G as one of the potential active components, and the high antioxidant activity observed in the ultrasonic extract, including the peel, suggests the potential contribution of non-flavonoid phenolic compounds with high-molecular-weight polyhydroxy structures, such as tannins, in addition to Q7G.

Although the limitations of the analytical method made it difficult to accurately quantify some high-molecular-weight antioxidant substances such as ellagitannins, this study pointed out their possible existence and suggested future analytical directions, thereby laying the foundation for future research. Furthermore, IU showed consistent efficacy, such as improved cell survival, NO production inhibition, and ROS reduction in zebrafish embryos, supporting actual biological functionality beyond simple antioxidant measurements.

## 5. Conclusions

In conclusion, this study goes beyond a simple solvent comparison study, providing practical evidence that removing the inner shell of QFs is not essential. It also proposes ultrasonic extraction as the most effective method for extracting functional active components under conditions that include the inner shell. This can serve as foundational data for achieving both efficient raw material utilization and maximized functionality in the development of QF-based functional foods and natural antioxidants. Additionally, it suggests a new research direction for reexamining the physiological activity potential of accessory tissues in other plant-based materials, including QF.

Our findings highlight that the QF inner shell is a valuable component rather than waste, and that an optimized extraction method can substantially enhance the functional utility of QF extracts. These insights offer a practical strategy for developing natural antioxidant ingredients and improving the utilization of plant materials in functional food production.

## Data Availability

Data is contained within the article.

## Supplementary Materials

The following supporting information can be downloaded at: https://www.mdpi.com/article/10.3390/antiox14080976/s1, Figure S1: Cell viability of Raw 264.7 cells treated with QF extracts.
