# Supplementary material for: Optimizing Antioxidant and Biological Activities of Quercus Fructus: Synergistic Role of Inner Shell and Extraction Methods
Source: Antioxidants (Basel). 2025 Aug 8;14(8):976. doi: 10.3390/antiox14080976 (PMC12382876; doi:10.3390/antiox14080976)
Supplement: Supplementary file 1 [file antioxidants-14-00976-s001.zip › antioxidants-3761507-supplementary.pdf]

Supplementary Figure S1

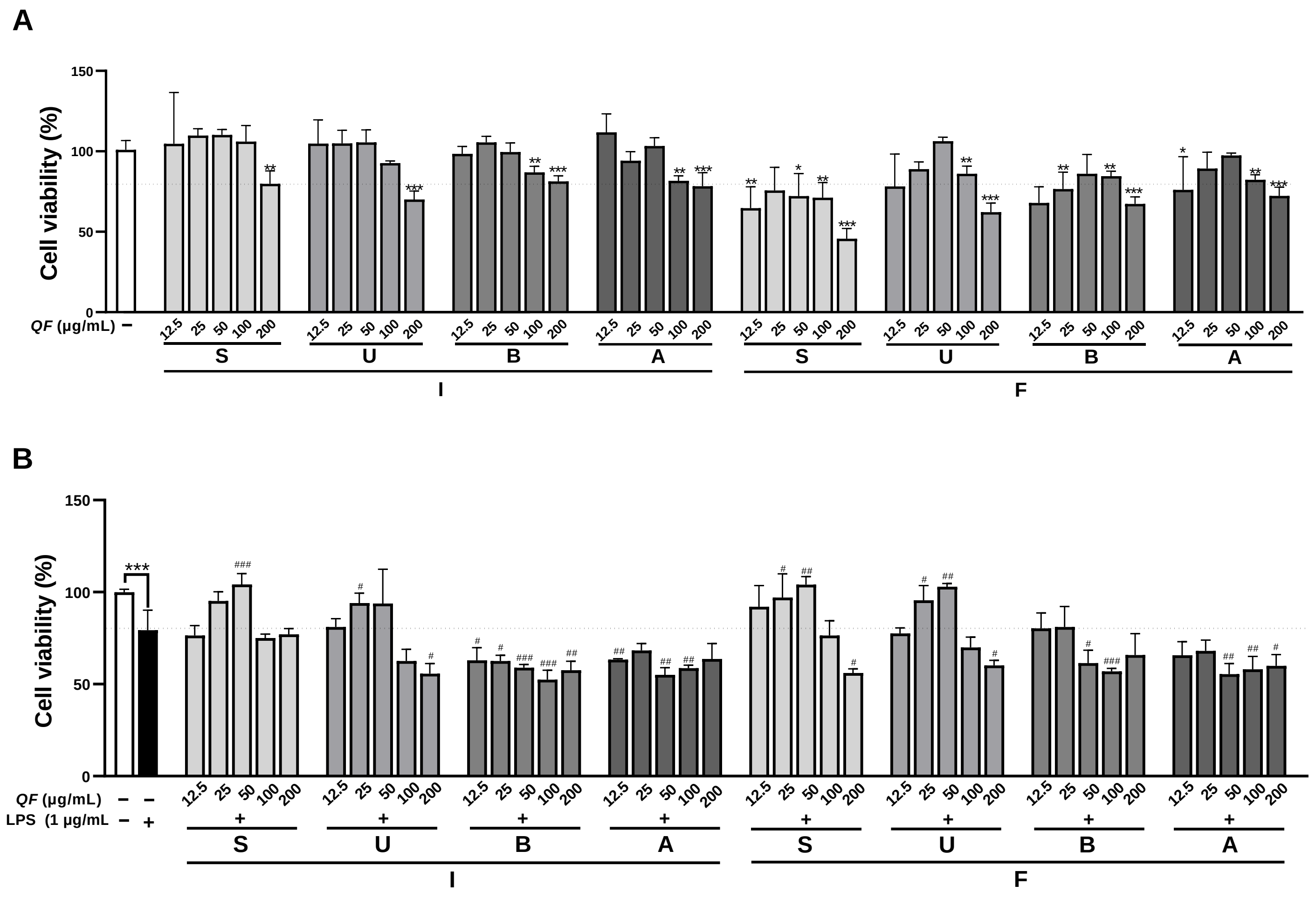

Figure S1. Cell viability of Raw 264.7 cells treated with QF extracts (S, U, B, A) for 48 hours in the presence or absence of LPS. Cell viability was measured in Raw 264.7 cells treated with different concentration of QF extracts for 48 hours in the absence (A) and presence of 1 μg/mL LPS (B). Results were represented as mean ± standard error with indication of statistical significance when the p-value is less than 0.05. The symbols are used to denote levels of significance (* $p < 0.05$, ** $p < 0.01$, *** $p < 0.001$, ## $p < 0.01$, ### $p < 0.001$; * denotes independent t-tests performed between negative control and experimental group; # denotes the results of an independent t-test between LPS treated positive control and experimental group; S: stirring, U: ultrasonication, B: boiled water, A: autoclave).
